# circVAR database: genome-wide archive of genetic variants for human circular RNAs

**DOI:** 10.1186/s12864-020-07172-y

**Published:** 2020-10-29

**Authors:** Min Zhao, Hong Qu

**Affiliations:** 1 School of Science and Engineering, University of the Sunshine Coast, Maroochydore DC, Queensland 4558 Australia; 2 Center for Bioinformatics, State Key Laboratory of Protein and Plant Gene Research, College of Life Sciences, Peking University, Beijing, 100871 P. R. China

**Keywords:** Bioinformatics database, Circular RNA, Genetic variant, Systems biology

## Abstract

**Background:**

Circular RNAs (circRNAs) play important roles in regulating gene expression through binding miRNAs and RNA binding proteins. Genetic variation of circRNAs may affect complex traits/diseases by changing their binding efficiency to target miRNAs and proteins. There is a growing demand for investigations of the functions of genetic changes using large-scale experimental evidence. However, there is no online genetic resource for circRNA genes.

**Results:**

We performed extensive genetic annotation of 295,526 circRNAs integrated from circBase, circNet and circRNAdb. All pre-computed genetic variants were presented at our online resource, circVAR, with data browsing and search functionality. We explored the chromosome-based distribution of circRNAs and their associated variants. We found that, based on mapping to the 1000 Genomes and ClinVAR databases, chromosome 17 has a relatively large number of circRNAs and associated common and health-related genetic variants. Following the annotation of genome wide association studies (GWAS)-based circRNA variants, we found many non-coding variants within circRNAs, suggesting novel mechanisms for common diseases reported from GWAS studies. For cancer-based somatic variants, we found that chromosome 7 has many highly complex mutations that have been overlooked in previous research.

**Conclusion:**

We used the circVAR database to collect SNPs and small insertions and deletions (INDELs) in putative circRNA regions and to identify their potential phenotypic information. To provide a reusable resource for the circRNA research community, we have published all the pre-computed genetic data concerning circRNAs and associated genes together with data query and browsing functions at http://soft.bioinfo-minzhao.org/circvar.

## Background

Circular RNAs (circRNA) are long non-coding RNAs (lncRNAs) that form covalently linked continuous loops and are abundant in eukaryotic cells [12]. circRNAs are generated by back-splicing events in which an upstream splice acceptor is linked to a downstream splice donor [12]. Most circRNAs have independent gene expression regulatory mechanisms which are different from their cognate linear forms. Rather than being regarded as by-products of transcription, there is emerging evidence that circRNAs play important regulatory roles at the transcriptional and post-transcriptional levels by acting as microRNA sponges and by modulating RNA binding protein genes [2]. Accumulated evidence revealed that circRNAs are associated with a broad range of diseases, including cancers, neurodegenerative diseases and cerebrovascular diseases [9]. These studies mainly focused on the expression of circRNAs, rather than the changes in DNA. For instance, circRNAs are generally down-regulated in comparison with corresponding normal tissues in cancer cells [9]. In general, these abnormal gene expression changes are primarily caused by genetic mutations that occur at the DNA level, and lead to altered interactions with mRNAs and proteins [11]. For example, the relationship between variants and expression in circRNA regions has been explored recently, which may provide an uncharacterized role of genetic changes in circRNA expression and regulation [1].

With the development of genome-wide array-based and sequencing technologies, millions of single nucleotide polymorphisms (SNPs) and small insertions and deletions (INDELs) have been linked to complex traits/diseases [4]. However, functional interpretation is challenging because many of these genetic changes are in non-coding regions. Considering the biological significance of circRNAs, we hypothesize that knowledge of genetic variations in poorly annotated circRNAs may provide insight into their roles in complex traits/diseases. To help identify putative circRNA-related SNPs and INDELs, we developed the first freely public available database, circVAR (http://soft.bioinfo-minzhao.org/circvar), which enables characterization of genetic variants in the human genome.

## Conclusion

Our primary goal is to identify the putative circRNA-related genetic SNPs and INDELs at the genome level. In current release, we did not explore the structural variants which might comprise multiple circRNAs. Therefore, it is not easy to evaluate the functional effects of a single circRNA from those hundreds of affected circRNAs. In sum, these pre-calculated genetic variants of circRNAs provide a comprehensive resource for discovering the commonality or uniqueness of genetic changes for all reported circRNAs. In sum, these pre-calculated genetic variants of circRNAs provide a comprehensive resource for discovery of the commonality or uniqueness of genetic changes for all reported circRNA. For example, previously studies have indicated that the protein-coding genes are unevenly distributed on 24 chromosomes, among which the densities of genes on chromosomes 1, 11 and 19 are particularly high [17]. Our circRNA distribution confirmed the high density of circRNAs on chromosome 19. Interestingly, we also found more clustered circRNAs on chromosome 17, which is different from the density of protein-coding genes.

The current version of circVAR contains: i) 93,708 annotated genetic variants with phenotype information from genome-wide associated studies (GWAS data from GWASCatalog); ii) 1,858,343 well-classified genetic variants with clinical applications from the ClinVAR database; iii) 2,597,987 somatic variants in cancer tissues from the COSMIC database; and iv) 26,361,367 common variants from the 1000 Genomes Project data. Our web interface also allows users to perform text queries and browse circRNAs based on their mapped genes and data sources. For advanced bioinformatics analysis, we have provided the bulk downloadable files for all the circRNAs with the two most popular genomic coordinates (GRCH 37 and GRCH 38). In addition, over 30 Gb of genetic variant annotation files were provided for the majority of the circRNAs.

Although the extensive integration and mapping of circRNA variants provides a blueprint for general genetic features, there are more circRNAs data generated from various tissues. Our goal is to incorporate more human circRNAs by curating the circRNAs from RNAseq data in the future. With the potential clinical and therapeutic applications of circRNAs, the genetic diversity in various human populations will become one of the keys to evaluate its risk. In addition, we may also conduct the more extensive meta-analysis on those circRNA-related variants with clinical phenotypes, because majority of GWAS hits are mapped in non-coding regions such as lncRNAs or circRNAs.

## Methods

### Data collection and processing

To provide a more comprehensive understanding of human circRNAs, we integrated three recent circRNAs databases: circBase [7], circNet [14] and circRNAdb [3]. These putative circRNA-related databases were built using large-scale genomic sequencing data. The circNet database contains the most comprehensive set of circRNAs, with 283,553 genomic locations, whereas circBase contains 92,375 coordinates and circRNAdb contains 32,914 coordinates. Genomic coordinates with circRNA evidence were downloaded. By using intersect command in Bedtools [15], all circRNA regions were mapped to each other. To remove redundancy, the minimum overlap required for any pair of circRNAs is higher than 90%. Due to the huge number of coordinates for the intersecting analysis, it is impossible to run all files in one job. To solve this computational challenge, we grouped circRNAs based on chromosome number. Using the high-performance computational facilities, we run the chromosome-based mappings one by one, which saved a lot of resources and improved the calculation accuracy.

In total, we collected 295,526 circRNAs based on GRCH37 genomic coordinates. By using the liftover toolkit from the UCSC genome browser [8], we also identified 295,073 unique circRNA coordinates in the GRCH 38 system [16]. The remaining 453 coordinates without mapping were discarded for the GRCH 38 version. For each circRNA with a unique chromosome location, we assigned a circVAR ID as the key for the database query.

### Annotation and database construction

To further annotate the genetic variant information for all integrated circRNAs, we downloaded four most popular human genetic resources, including the 1000 Genomes Project (downloaded from the 1000 genomes FTP, v5b.20130502) [6], GWASCatalog (downloaded July 18, 2017) [18], ClinVAR (downloaded July 18, 2017) [10], and COSMIC V81 [5].

For those single nucleotide variants, small insertions and deletions, we directly run intersect command in Bedtools to map all variants to any circRNA region. Since we have 295,526 circRNA coordinates to be intersected with those genetic variant data from various databases, and there are thousands of millions of variants in those public databases, the computational cost of these mappings is very high. Among the four variant databases that we are going to map, the 1000 Genome Project contains the largest number of variants. The recently released 1000 Genome Project is the phase 3 release with 84.4 million variants from 26 populations. Multiplying our 295,526 circRNA coordinates will require 24,942,394 million (approximately 25 trillion) intersection operations, which requires a lot of memory, CPU time and read/write operations on disks. Especially, it is not feasible to load all 84.4 million variants into memory for overlapping analysis. Therefore, we implemented our calculation by dividing the data into multiple pieces based on the chromosome number. For example, we used the intersect command in Bedtools, as shown below, to find out the potential overlap between circRNAs on chromosome 1 and 1000 Genome data: bedtools intersect -a circRNA_chromosome1.bed -b 1000genome.bed -wa -wb. Finally, the chromosome-based mappings were submitted to the high-performance computing system.

All mapped results were exported as plain text and directly imported to the database system under Linux server. In order to manage our data efficiently, we constructed the relational database by using MYSQL system. In our database, all the original circRNA IDs were recorded to allow results to be traced back to the original circRNA resource and literature reports. Since the functions of circRNAs are primarily related to their neighbour genes, we collected all neighbour protein-coding genes for all circRNAs based on their genomic locations. In summary, by linking predefined circRNAs with genetic variants, literature reports and protein-coding genes, we provided information regarding the potential functional associations of circRNAs.

### Web interface to search and browse data

**Fig. 1:**
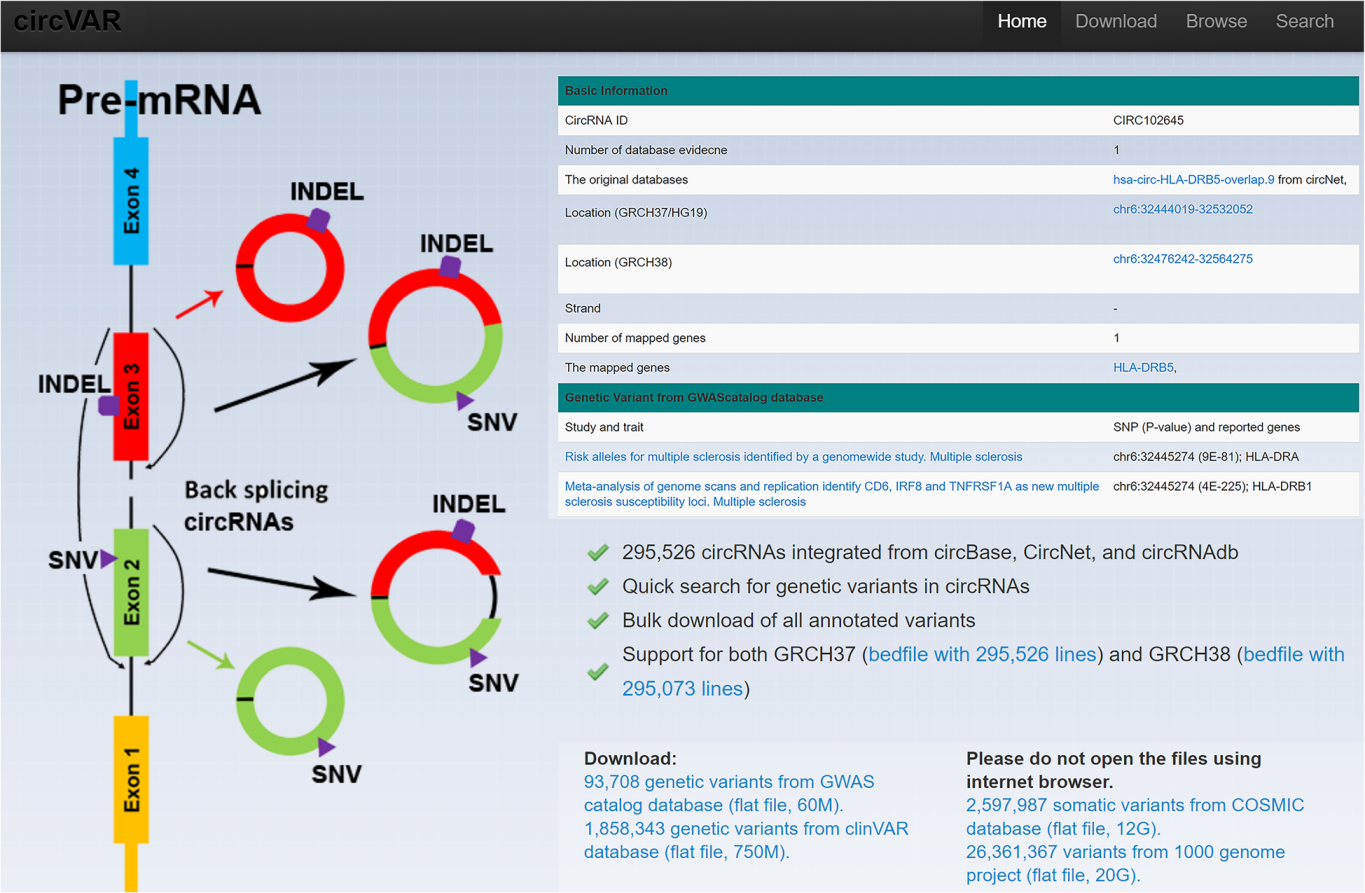
Web interface of the circVAR database. In this version, we focused on single nucleotide variations and small insertions and deletions (INDELs). The page implemented data downloading, browsing and searching. For each circRNA in the database, the gene information is shown on the top right. In total, we integrated 295,526 circRNAs based on the GRCH 37 coordinates from circBase, circNet, and circRNAdb. By mapping to GRCH 38 coordinates, we identified 295,073 circRNAs. The basic information for each circRNA includes the circRNA ID in our database, data source, genomic locations for both GRCH 37 and GRCH 38, mapped genes and pre-computed genetic variants from GWASCatalog, ClinVAR, COSMIC, and the 1000 Genomes Project. Due to the huge volume of data, we provided bulk downloading for all pre-computed genetic variants from the four genetic variant databases

To ensure that the pre-computed data was widely accessible, we implemented a web server for data searching and browsing based on genomic location, a cancer-related gene list, and different data sources (Fig. 1). A typical circRNA entry contained eight information categories: circRNA ID in our database; data source; genomic locations for both GRCH 37 and GRCH 38; mapped genes; and pre-computed genetic variants from four databases. For the pre-computed variants associated with GWAS studies, we presented detailed phenotype information. Due to the huge task of mapping to ClinVAR, COSMIC and 1000 Genomes data, we linked all genetic variants to the original databases. For the large-scale integrative analyses, we provided the bulk data by downloading all circRNAs-associated variants. As presented in the downloading page, the data files are: i) 60 Mb for 93,708 genetic variants from the GWASCatalog database; ii) 750 Mb for 1,858,343 genetic variants from the ClinVAR database; iii) 12Gb for 2,597,987 somatic variants from the COSMIC database; iv) 20Gb for 26,361,367 variants from the 1000 Genomes Project.

circVAR allows a number of ways to browse putative circRNAs and associated genetic variants, including chromosome distribution, associated genes, data sources for circRNAs and data sources for genetic variants. The genomic locations of all circRNAs have been plotted on 24 chromosomes. Users can browse each chromosome to access all the circRNAs in the region. The critical cancer driver genes associated with each circRNA are provided, indicating their relationship to oncogenes [13], tumor suppressors [19], and cancer metastasis-related genes [13]. To provide access to different data sources for the circRNAs and genetic variants, we included a browsing function that identifies different circRNA and genetic variant databases.

Three search functions were implemented to permit searches of circRNAs, associated genes, and genetic variants. To conduct rapid queries about circRNAs, users can input IDs as follows: CIRC102645 (ID from circVAR), hsa-circ-A2M.21 (ID from circNET), hsa_circ_0013273 (ID from circBase), or hsa_circ_09535 (ID from circRNAdb). Associated gene searches can be conducted by typing the gene name or its Entrez gene ID. In addition, users can search all pre-computed genetic variants by specifying the corresponding database and IDs, including COSM5035133 (Mutation ID from the COSMIC database), 14,637 (variation ID from the Entrez clinVAR database), rs1505368 (dbSNP ID from the GWASCatalog and 1000 Genomes data). This technique is useful in identifying candidate circRNAs for specific genetic mutations.

## Results and discussion

### Chromosome distribution of circRNAs

**Fig. 2:**
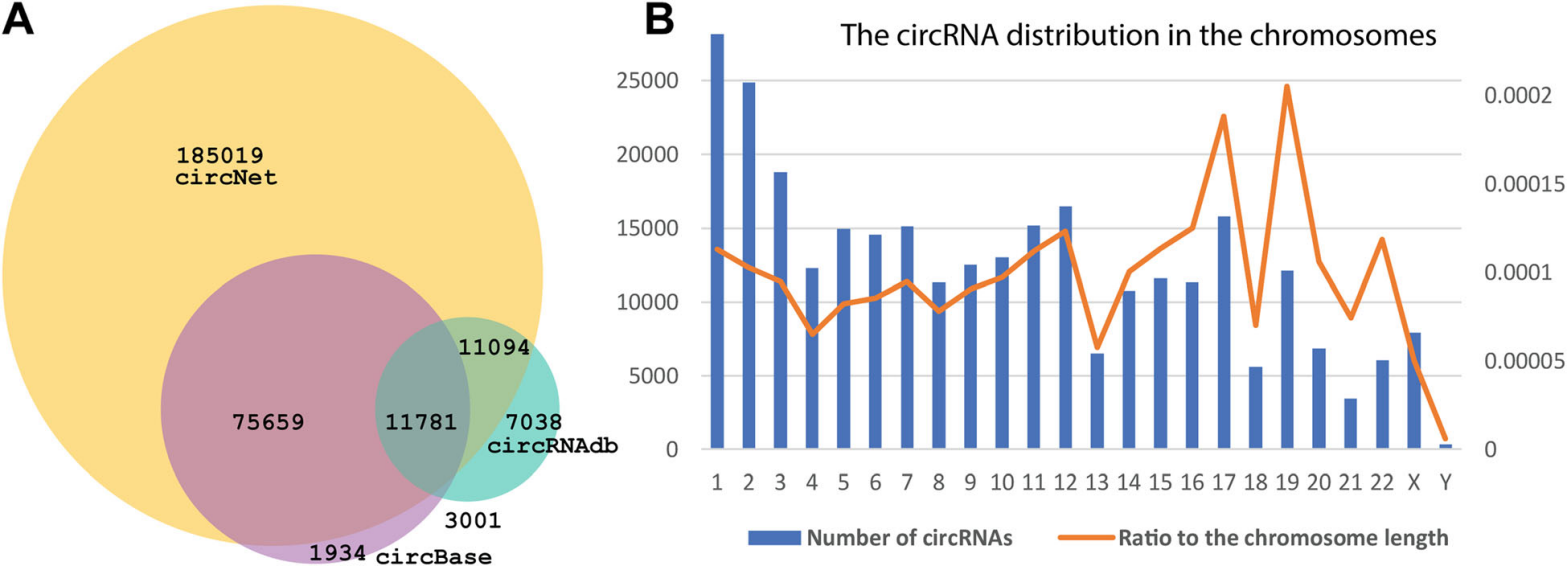
Data integration and chromosome distribution of human circRNAs. **a** The overlapping relationship of the three integrated human circRNA datasets based on the exact chromosome location. **b** The chromosome distribution of all unique circRNAs. The number of circRNAs in each chromosome is shown in the blue bar chart. The orange line is the ratio between the number of circRNAs and the length of the chromosome

Based on all three data sources, we defined the unique circRNA sets in our database according to their genomic location and strand. The three datasets shared a total of 11,781 circRNA records (Fig. 2a). The circNet database was the largest database, with 185,019 unique circRNA records, while circBase had the fewest specific records (1934 circRNAs). In total, 101,535 circRNAs were validated using two or more data sources. For instance, circNet and circBase shared 75,659 circRNA records, providing cross-validation. In summary, our integration re-visited current public data sources for circRNAs and provided a non-redundant circRNA list for experimental verification.

To explore circRNAs in different chromosomes, we plotted the numbers of all circRNAs across all chromosomes and calculated the ratio of circRNAs using chromosome length (base pairs) as the basis (Fig. 2b). The largest chromosome, chromosome 1, has the most circRNAs records (28,161). To confirm which chromosomes have enriched circRNAs, we calculated the ratio between the number of circRNAs and the chromosome length. Interestingly, we found that chromosome 17 and 19 have a comparatively higher number of circRNAs, although these chromosomes are relatively short. In contrast, chromosome X is large, but has only 7927 circRNAs with a small ratio of 0.00005. As the first comprehensively integrated human circRNA resource, the chromosome distribution of the circRNAs in our database provides insight into the abundance of circRNAs in different genomics regions, which is valuable for linking known genomics events and processes.

### Linking circRNA variants to population frequency and phenotype by overlapping with genetic variant databases

In order to link those circRNA variants with phenotype information, such as clinical significance and allele frequency in various populations, we downloaded all single nucleotide mutations and INDELs based on the GRCH 37 coordinates from four resources: the 1000 Genomes Project, GWASCatalog, ClinVAR, and COSMIC V81. By using genome mapping algorithm, we identified those SNPs/INDELs within the chromosomal locations of those integrated circRNAs. Based on the coordinate mapping, we yielded: i) 37,399 circRNAs with 93,708 genetic variants from GWAScatalog; ii) 67,661 circRNAs with 1,858,343 genetic variants from clinVAR; iii) 236,762 circRNAs with 2,597,987 somatic variants from COSMIC; and iv) 291,729 circRNAs with 26,361,367 variants from the 1000 Genomes Project.

**Fig. 3:**
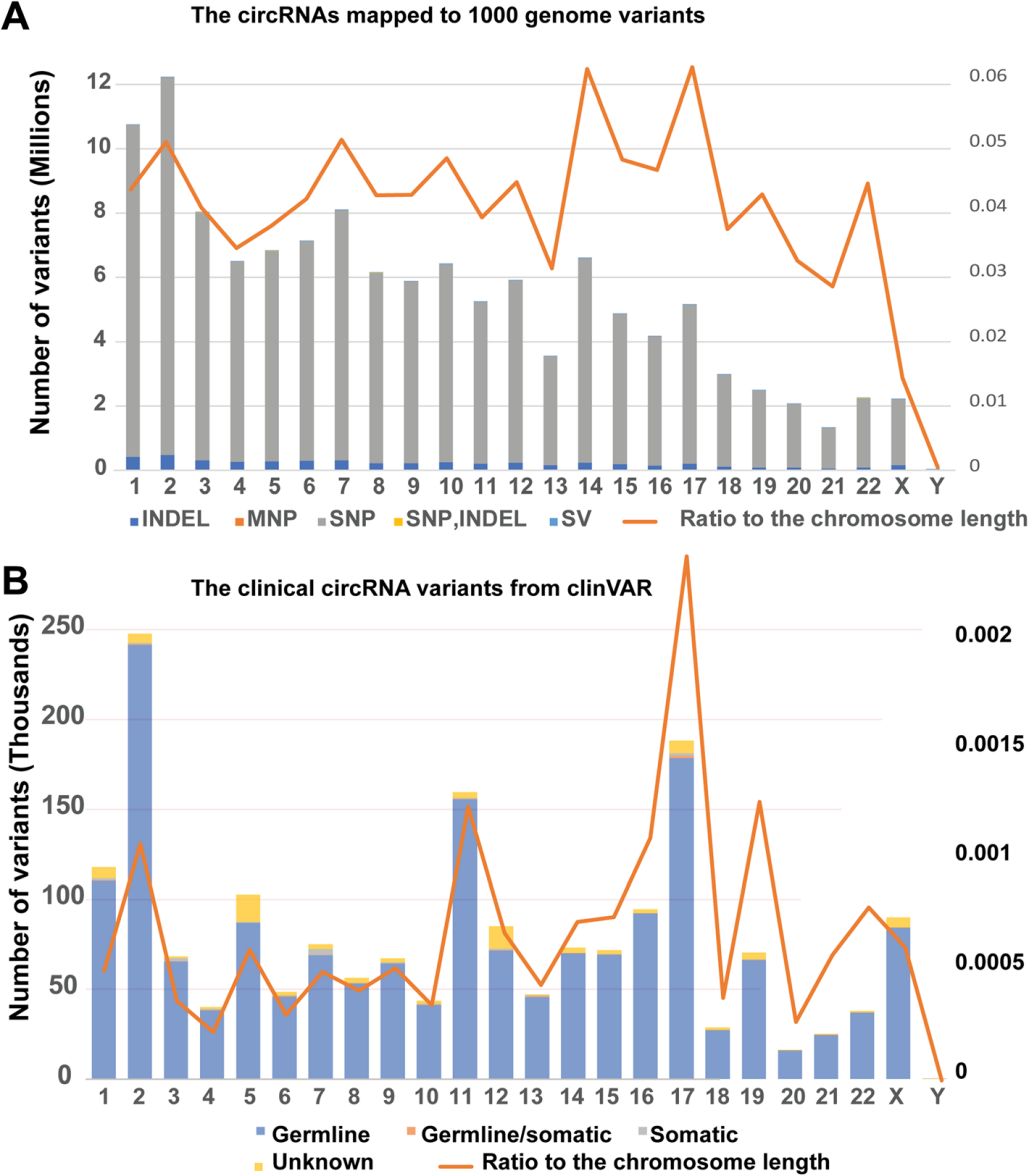
circRNAs mapped to the 1000 Genomes Project and the clinVAR database. **a** The chromosome distribution of common variants from the 1000 Genomes Project. SNP: single nucleotide polymorphism; MNP: multiple nucleotide polymorphism; INDEL: insertion and deletion; SV: structure variant. The orange line is the ratio between the number of circRNA variants from the 1000 Genomes Project and the length of the chromosome. **b** The chromosome distribution of circRNA variants with phenotypes from the clinVAR database. The orange line is the ratio between the number of circRNA variants from the ClinVAR database and the length of the chromosome

Since the genetic variants from the 1000 Genomes Project are mostly common variants in the healthy population [6], the distribution of circRNA variants in the chromosome may provide a population-based overview. To explore this possibility, we plotted all circRNA-associated common variants from different chromosomes and checked the ratio for these variants by benchmarking in relation to chromosome length. As shown in Fig. 3a, chromosome 2 has the highest number of common variants associated with circRNAs (12,232,567), followed by chromosome 1 with 10,765,543. Since the majority of the variants are at the single nucleotide level, SNPs overlapping with circRNAs may have a neutral or minor effect with regard to changing transcript information. However, thousands of large-scale structure variants (SV) were detected in the circRNA regions. For example, circRNAs on chromosome 2 overlap with 7294 SVs. SVs can change nucleotides and the number of gene copies of circRNAs which, in turn, may have a large effect on transcription expression status. Chromosome 17 is short, but its circRNAs have a comparatively large number of variants. In summary, mapping to the 1000 Genomes data for circRNAs may help users to understand the population-based genetic frequency of common variants within circRNAs.

The clinVAR database aims to aggregate genetic variation and its relationship to human health. Therefore, changes in circRNA regions may have significant effects on cellular process and, as shown in Fig. 3b, the majority of the variants are germline and contribute to various clinical phenotypes. The circRNA variants from clinVAR on chromosome 2 have the most abundant circRNA variants related to human health. However, chromosome 1 has half as many clinical variants as chromosome 2. In addition, chromosome 17 has the highest clinical variants: chromosome length ratio. It is worth noting that most of the clinical variants are based on studies of protein-coding regions. Since circRNAs have many regions overlapping with their corresponding protein-coding transcripts, changes in circRNAs may profoundly affect gene expression.

### Variant types of circRNAs in the human genome

**Fig. 4:**
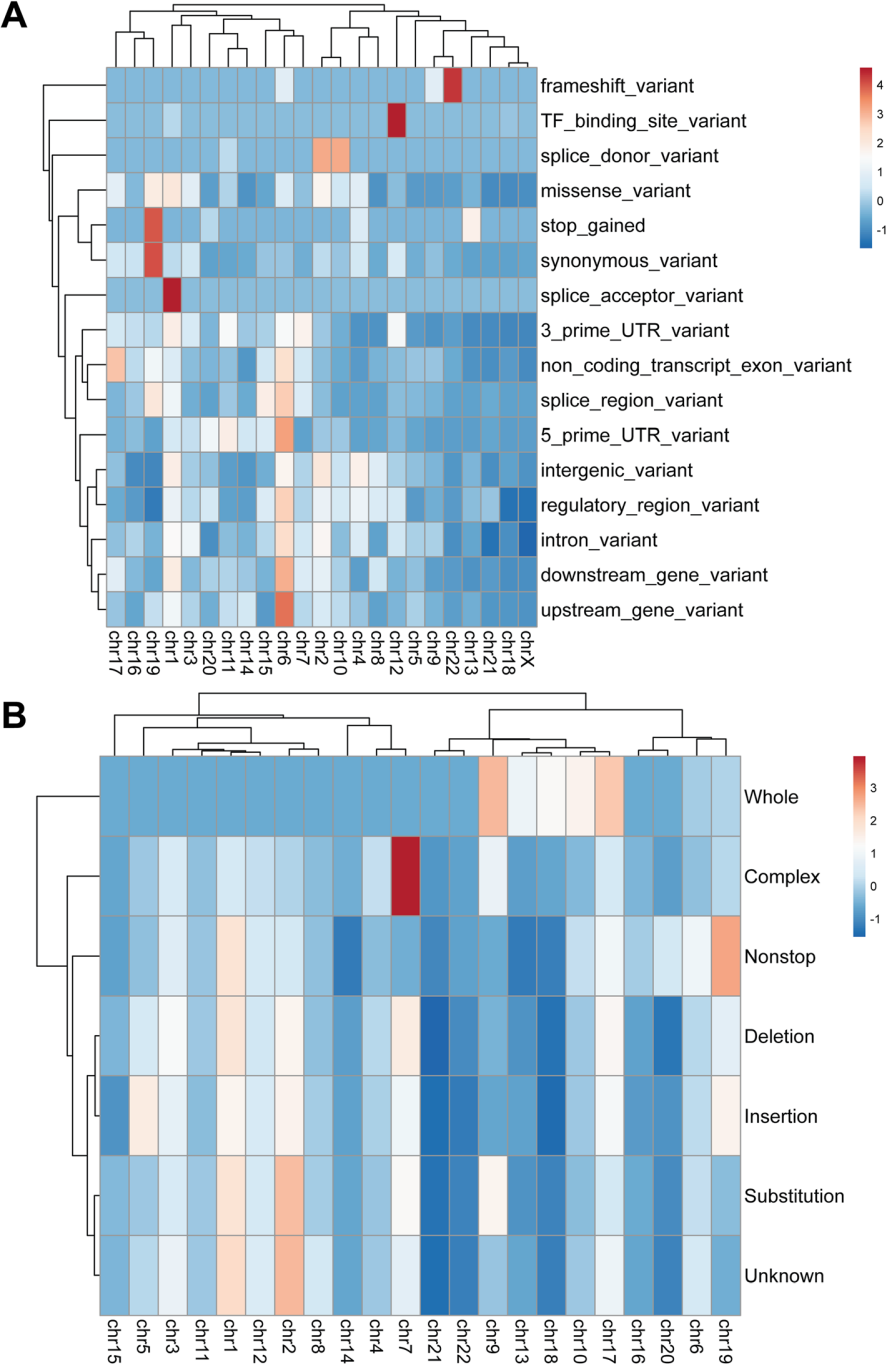
The circRNA variants from GWAS and cancer genomics studies. **a** The circRNAs were mapped to the GWASCatalog to annotate their potential association with phenotypes. The Z-scores were transformed from the numbers for a specific variant type across different chromosomes. The polarity of the Z-score reflects whether the result is higher or lower than the average. **b** The circRNAs were mapped to the COSMIC database to annotate their potential association with various cancers. The Z-scores were transformed from the numbers for a specific variant type across different chromosomes. The polarity of the Z-score reflects whether the results is higher or lower than the average

To discover which genetic variants are more likely to occur in the circRNA regions, we overlapped the circRNAs with the GWASCatalog and COSMIC databases. GWASCatalog contains the records of all published genetic variants with phenotype information from large scale genome-wide association studies. The COSMIC database is primarily focused on cancer-related somatic mutations. For all genetic variants, we used the effects described in the original database to define their type. For example, there are 16 categories in the GWASCatalog, including three prime UTR variants and a transcription factor binding site (Fig. 4a). The data from COSMIC grouped the variants into seven types: whole, complex, deletion, insertion, nonstop, substitution, and unknown (Fig. 4b). By combining the genomic location and variant for each circRNA variant, we constructed a matrix to present the number of specific variant types in a given chromosome. Based on these numbers, we performed Z-transformation to identify those that were significantly higher or lower than the average.

Based on the data mapped to GWASCatalog, we found that a large number of circRNA variants were not within protein-coding regions. For example, chromosome 1 has 4754 introns and 1836 intergenic circRNA variants. In fact, the intron and intergenic variants were evenly distributed in all chromosomes (Fig. 4a). However, there were a number of chromosomes with large Z-scores, indicating that they contain a higher than average number of circRNA variants. For instance, chromosome 12 has 33 circRNA variants located in the transcription factor binding region, which is the greatest number found among the chromosomes. As the fundamental regulatory mechanism, transcription factor binding affects protein coding gene expression and has profound effects on circRNA expression. In summary, over 90% of circRNA variants from the GWASCatalog belong to non-coding regions. In addition, these non-coding circRNA variants are spread equally among all chromosomes. Unlike mutations in coding regions, non-coding changes in circRNAs may have profound effects on the expression of circRNAs, suggesting novel mechanisms for common diseases.

Using variants from the cancer datasets (Fig. 4b), we observed an even distribution of cancer-related somatic variants across multiple chromosomes. Chromosome 2 has the highest number of cancer-related variants: 3,246,573. This finding is not surprising, as the majority of somatic variants are of the substitution type. For example, there are 2,605,118 substitutions among a total of 2,810,771 variants on chromosome 1. It is important to note that chromosome 7 has a total of 10,756 complex mutations compared to the average of 2225 in all chromosomes. Complex mutations have multiple insertions, deletions and substitutions. The huge number of complex mutations may provide driver mechanisms that influence the expression of circRNAs.

## Data Availability

All the data is free to use for academic purpose at http://soft.bioinfo-minzhao.org/circvar.

## Abbreviations

circRNAs: circular RNAs
INDEL: small insertion and deletion
SNP: single nucleotide polymorphism
lncRNAs: long non-coding RNAs
circVAR: genetic variant database for human circular RNAs
circBase: a database for circular RNAs
circNet: a database of circular RNAs derived from transcriptome sequencing data
circRNAdb: a comprehensive database for human circular RNAs with protein-coding annotations
The 1000 Genomes Project: a deep catalog of human genetic variation from the 1000 Genomes Project
ClinVAR: a public archive of reports of the relationships among human variations and phenotypes
COSMIC: the Catalogue of Somatic Mutations in Cancer
GWAS: Genome-Wide Association Studies
GWAS Catalog: a curated collection of all human genome-wide association studies
